# Sensing Attribute Weights: A Novel Basic Belief Assignment Method

**DOI:** 10.3390/s17040721

**Published:** 2017-03-30

**Authors:** Wen Jiang, Miaoyan Zhuang, Chunhe Xie, Jun Wu

**Affiliations:** School of Electronics and Information, Northwestern Polytechnical University, Xi’an 710072, China; zhuang-my@mail.nwpu.edu.cn (M.Z.); xiechunhe@mail.nwpu.edu.cn (C.X.); junwu@nwpu.edu.cn (J.W.)

**Keywords:** Dempster–Shafer evidence theory, generalized evidence theory, basic belief assignment, soft sensors data fusion, Gaussian distribution, attribute weights

## Abstract

Dempster–Shafer evidence theory is widely used in many soft sensors data fusion systems on account of its good performance for handling the uncertainty information of soft sensors. However, how to determine basic belief assignment (BBA) is still an open issue. The existing methods to determine BBA do not consider the reliability of each attribute; at the same time, they cannot effectively determine BBA in the open world. In this paper, based on attribute weights, a novel method to determine BBA is proposed not only in the closed world, but also in the open world. The Gaussian model of each attribute is built using the training samples firstly. Second, the similarity between the test sample and the attribute model is measured based on the Gaussian membership functions. Then, the attribute weights are generated using the overlap degree among the classes. Finally, BBA is determined according to the sensed attribute weights. Several examples with small datasets show the validity of the proposed method.

## 1. Introduction

Data fusion technology of soft sensors [1,2,3] which integrates multi-source information to obtain a more objective result, is widely used in many application systems [4,5,6]. Some algorithms, such as fuzzy sets theory [7,8,9,10], Dempster–Shafer evidence theory (D-S evidence theory) [11,12], Z numbers [13,14], D numbers [15,16,17] and neural networks [18,19,20,21,22,23,24], are important tools in the data fusion of soft sensors. As one of them, D-S evidence theory gives a convenient mathematical framework to handle uncertainty information, so it is widely used in many fields [25,26,27]. However, determination of basic belief assignment (BBA) is the first and key step for applying D-S evidence theory. In general, there is no fixed model to obtain BBA. The method is usually designed according to the practical application.

Many scholars have investigated different methods to address the problem of obtaining BBA. Yager [28] proposed the D-S belief structure to determine BBA using the whole class of fuzzy measures. Zhu et al. [29] used fuzzy c-means to obtain BBA. Dubois et al. [30] proposed a probability–possibility transformation to gain BBA. Baudrit and Dubois [31] proposed practical representation methods for incomplete probabilistic information, based on formal links existing between possibility theory, imprecise probability and belief functions. Wang et al. [32] derived BBA function from the common multivariate data spaces. Masson and Denoeux [33] constructed a possibility distribution from a discrete empirical frequency distribution. Antoine et al. [34] constrained evidential clustering with instance-level constraints to determine BBA. Campos and Huete [35] determined BBA by considering the problem of assessing numerical values of possibility distributions.

Although various methods are proposed, these methods mainly focus on the incompleteness or the uncertainty of information itself, which does not simultaneously consider the importance and reliability of information sources. In the real world, information is not only incomplete and fuzzy, but also partly reliable. These are all the embodiment of the uncertainty of the information. We cannot only consider the ambiguity of information without considering the reliability of the information itself. For instance, in multiple attribute decision making, the importance of each attribute is different, so the weight of BBAs which are generated by different attributes is different. Many methods are proposed for measuring the weight of BBAs in D-S evidence theory [36,37]. The methods to determine these BBAs mainly depend on the support degree among BBAs, which are driven by data and relatively objective, however, these methods are questionable. The importance and reliability of these BBAs are not measured from the source of BBAs. Traditionally, the reliability of the information source is mainly evaluated based on the subjective judgement of the domain experts. However, the knowledge of experts is limited, which would lead to unreasonable results before some effective analysis is done. So far, the existing methods to determine BBA cannot address this issue, both in the closed world and in the open world. To address this issue, a method to determine BBA is proposed based on the attribute weights. The reliability of the information source is taken as a factor to correct the traditional BBA, and then BBA is gained which contains the reliability of the information source. The advantage of this method is that the reliability of BBA is measured by the attribute weights, which are driven by data and more reasonable.

Many studies have shown that unreasonable results may appear when using the classical D-S evidence theory in the open world. Based on this issue, Smets and Kennes [38] proposed the transferable belief model (TBM), and firstly introduced the concepts of the closed world and the open world, which has been widely applied in many fields [39,40,41]. The advantage of the TBM is that it holds belief at two levels, namely the credal level and the pignistic level. However, regardless of the credal level or pignistic level, the empty set is not assigned belief value in the BBA generation phase. Instead, the empty set is seen as an alarm or intermediary to show that conflict exists which may be caused by an incomplete framework of discernment in the process of evidential reasoning. As a result, the belief is assigned to the empty set in the process of evidential reasoning if the open world assumption is held, but not at the stage of BBA generation. Hence, Deng [42] presented the generalized evidence theory (GET), where generalized basic belief assignment (GBBA) and generalized combination rule (GCR) are proposed. GET assumes the decision-making environment is in an open world at the initial phase, so a generalized BBA that allows the beliefs to be assigned to the empty set is generated at the stage of data collection and modelling, which is more reasonable. GBBA will degenerate to classical BBA when m(∅)=0. Therefore, in this paper, a new method to determine BBA in the open world is presented based on GET. However, the GCR in GET still has some problems. For example, the method of obtaining m(∅) in the GCR is unreasonable. Based on this issue, a modified generalized combination rule (mGCR) in the framework of GET is presented [43]. The mGCR satisfies all properties of the GCR and is more reasonable than the GCR.

In this paper, based on the attribute weights, BBA is determined not only in the closed world, but also in the open world. First, the Gaussian distribution is used to model the Gaussian membership function of each attribute of each class. Second, the similarity between the test sample and the attribute model is measured based on these Gaussian membership functions. Then, the attribute weights are generated using the overlap degree among the classes. Given an attribute, the larger the overlap degree, the smaller the attribute weight; conversely, the smaller the overlap degree, the larger the attribute weight. Finally, BBA is determined after modifying the similarity by the sensed attribute weights. This BBA contains not only the information of each class, but also the reliability of each attribute, which is more objective and reasonable.

The rest of this paper is organized as follows. D-S evidence theory and some necessarily related concepts are briefly presented in Section 2. In Section 3, a novel method of determining BBA based on attribute weights is proposed. Several examples are illustrated in Section 4 to show the efficiency of this method. The conclusion is presented in Section 5.

## 2. Preliminaries

### 2.1. Dempster–Shafer Evidence Theory [11,12]

D-S evidence theory was introduced by Dempster. Then Shafer developed it by defining the belief function and plausibility function. It has the ability to deal with uncertain information without a prior probability. Thus, it is flexible and more effective than probability theory. Due to these advantages, it is widely applied in many fields [44,45,46].

#### 2.1.1. Frame of Discernment (FoD) and Mass Function

In D-S evidence theory, the frame of discernment (FoD) is defined as $\Omega =\{H_{1},H_{2},\cdots ,H_{N}\}$, which is composed of *N* exhaustive and exclusive hypotheses. Denote P(Ω), the power set which is composed with $2^{\Omega }$ propositions as:

(1) $$P\left(\Omega \right)=\{\emptyset ,\left\{H_{1}\right\},\left\{H_{2}\right\},\cdots ,\left\{H_{N}\right\},\left\{H_{1}\cup H_{2}\right\},\left\{H_{1}\cup H_{3}\right\},\cdots ,\Omega \},$$

where the propositions $\left\{H_{1}\right\},\left\{H_{2}\right\},\cdots ,\left\{H_{N}\right\}$ have only one element, so they are defined as the singleton subsect proposition; the propositions $\left\{H_{1}\cup H_{2}\right\},\left\{H_{1}\cup H_{3}\right\},\cdots ,\Omega$ have more than one element, so they are defined as the compound subsect proposition.

A mass function *m* is a mapping from $2^{\Omega }$ to [0,1], formally defined as

(2) $$m:2^{\Omega }\to \left[0,1\right],$$

which satisfies the following conditions:

(3) $$\begin{array}{c} \sum_{A\subset 2^{\Omega }}m\left(A\right)=1\\ m(\emptyset )=0. \end{array}$$

The mass function *m* is also called BBA function. Any subset A of Ω such that m(A)>0 is called a focal element.

#### 2.1.2. Dempster’s Combination Rule

Suppose $m_{1}$ and $m_{2}$ are two mass functions in the same FoD Ω. Dempster’s combination rule, noted by $m=m_{1}⊕m_{2}$, is defined as follows:

(4) $$m\left(A\right)=\left\{\begin{array}{cc} \frac{\sum_{B\cap C=A}m_{1}\left(B\right)m_{2}\left(C\right)}{1-k}, & A\neq \emptyset \\ 0, & A=\emptyset \end{array}\right.$$

where

(5) $$k=\sum_{B\cap C=\emptyset }m_{1}\left(B\right)m_{2}\left(C\right).$$

Here, *k* is regarded as a measure of conflict between $m_{1}$ and $m_{2}$. The larger the value of *k*, the more conflict between the evidence.

### 2.2. Generalized Evidence Theory

Generalized evidence theory (GET) is presented by Deng, which can handle the uncertain information in the open world [42].

#### 2.2.1. Generalized Basic Belief Assignment

Suppose that *U* is a FoD in the open world. Its power set, $2_{G}^{U}$, is composed of $2^{U}$ propositions. A mass function is a mapping $m_{G}:2_{G}^{U}\to \left[0,1\right]$ that satisfies

(6) $$\sum_{A\subset 2_{G}^{U}}m_{G}\left(A\right)=1,$$

where $m_{G}$ is GBBA of the *U*.

The difference between GBBA and classical BBA is the restriction of ∅. Note that $m_{G}\left(\emptyset \right)=0$ is not necessary for GBBA, namely, the empty set can also be a focal element. GBBA will degenerate to classical BBA when m(∅)=0.

#### 2.2.2. Generalized Combination Rule

In GET, $\emptyset_{1}\cap \emptyset_{2}=\emptyset$ is assigned to conflict coefficient *K*. Given two GBBAs ($m_{1}$ and $m_{2}$), the GCR is defined as follows:

(7) $$\begin{array}{c} m\left(A\right)=\frac{\left(1-m\left(\emptyset \right)\right)\sum_{B\cap C=A}m_{1}\left(B\right)m_{2}\left(C\right)}{1-K},\\ K=\sum_{B\cap C=\emptyset }m_{1}\left(B\right)m_{2}\left(C\right),\\ m\left(\emptyset \right)=m_{1}\left(\emptyset \right)\cdot m_{2}\left(\emptyset \right),\\ m(\emptyset )=1\;\;\;if\;and\;only\;if\;\;\;K=1. \end{array}$$

### 2.3. Modified Generalized Combination Rule

The mGCR is presented by Jiang et al., where the intersection $\emptyset =\emptyset_{1}\cap \emptyset_{2}$ is considered as support to ∅, namely, the orthogonal sum of $m_{1}\left(\emptyset \right)$ and $m_{2}\left(\emptyset \right)$ is normalized like other focal elements. Given two GBBAs, the mGCR is defined as follows:

(8) $$\begin{array}{c} m\left(A\right)=\frac{\sum_{B\cap C=A}m_{1}\left(B\right)m_{2}\left(C\right)}{1-K}\\ m\left(\emptyset \right)=\frac{m_{1}\left(\emptyset \right)m_{2}\left(\emptyset \right)}{1-K} \end{array}$$

with

(9) $$\begin{array}{c} K=\sum_{\begin{array}{c} B\cap C=\emptyset \\ B\cup C\neq \emptyset \end{array}}m_{1}\left(B\right)m_{2}\left(C\right)\\ m\left(\emptyset \right)=1\;\;\;if\;K=1\;or\;\sum_{A\neq \emptyset }m\left(A\right)=0. \end{array}$$

The mGCR inherits all the advantages and properties of the original GCR.

## 3. The Proposed Method

**Figure 1:**
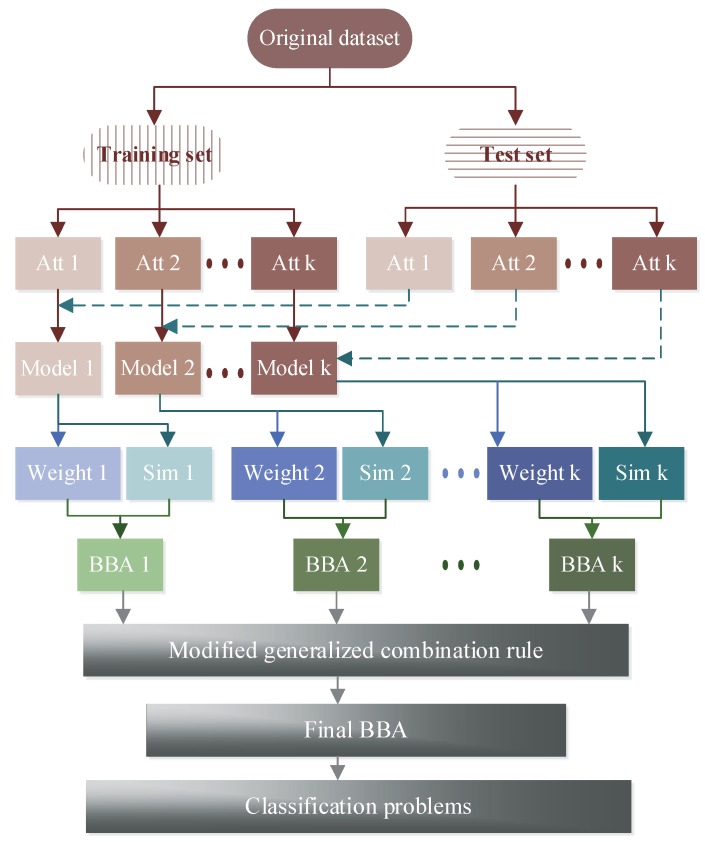
Flowchart of the proposed method.

To obtain an objective classification, the key step is how to gain an effective BBA. As can be seen in Figure 1, a new method to determine BBA is presented. First, the dataset is divided into two parts, where a part is taken as the training set and another part is taken as the test set. Then the Gaussian models of *k* attributes are built using the training set, and the attribute models are tested by the test set to produce the similarity. Finally, the weight of each attribute is constructed based on the overlap degree of each class, which is used to modify the similarity and then to determine BBA.

### 3.1. The Modeling of Each Attribute

The Gaussian distribution is the common probability distribution in statistics, which is easy to analyze. Therefore, the membership function of Gaussian type is adopted to build the attribute models in this paper.

Suppose in the frame of discernment (FoD) $\Theta =\{\theta_{1},\theta_{2},\cdots ,\theta_{n}\}$, each class $\theta_{i}\;\left(i=1,2,\cdots ,n\right)$ has j(j=1,2,⋯,k) attributes. The mean value ${\bar{X}}_{ij}$ and the standard deviation $\sigma_{ij}$ of all the training samples in class $\theta_{i}$ are calculated as follows:

(10) $$\begin{array}{c} {\bar{X}}_{ij}=\frac{1}{N}\sum_{l=1}^{N}x_{ijl},\\ \sigma_{ij}=\sqrt{\frac{1}{N-1}\sum_{l=1}^{N}{\left(x_{ijl}-{\bar{X}}_{ij}\right)}^{2}}, \end{array}$$

where $x_{ijl}$ is the sample value of the *j*th attribute from the *l*th training sample in class $\theta_{i}$.

Hence, the corresponding attribute model of Gaussian type is obtained as follows:

(11) $$\mu_{A}\left(x\right)=exp\left(-\frac{{\left(x-{\bar{X}}_{ij}\right)}^{2}}{2\sigma_{ij}^{2}}\right)$$

The real-world sensor data usually has stochastic nature. To make the results more objective and credible, the training samples from each class are randomly selected to build the attribute model. For each attribute, *n* membership functions of Gaussian type are obtained as models of different class in the specific attribute.

#### 3.1.1. The Construction of the Singleton Subsect and Compound Subsect

In D-S evidence theory, BBA has two forms. One is the singleton subsect, such as {A}, which represents a certain proposition. The other is the compound subsect, such as {AB}, which represents a kind of uncertain proposition. Namely, it indicates that {A} proposition or {B} proposition occurs, and it is unknown how to assign belief in {A} and {B}. For instance, in the case of motor rotor fault diagnosis, there are three faults: A, B and C, which represent the unbalance, misalignment and pedestal looseness respectively. BBA from a sensor is m({A})=0.55,m({AC})=0.45. Where {A} represents that the fault of the motor rotor is the unbalance, which is a singleton subsect proposition; {AC} represents that the fault of the motor rotor is the unbalance or pedestal looseness, which is a compound subsect proposition.

**Figure 2:**
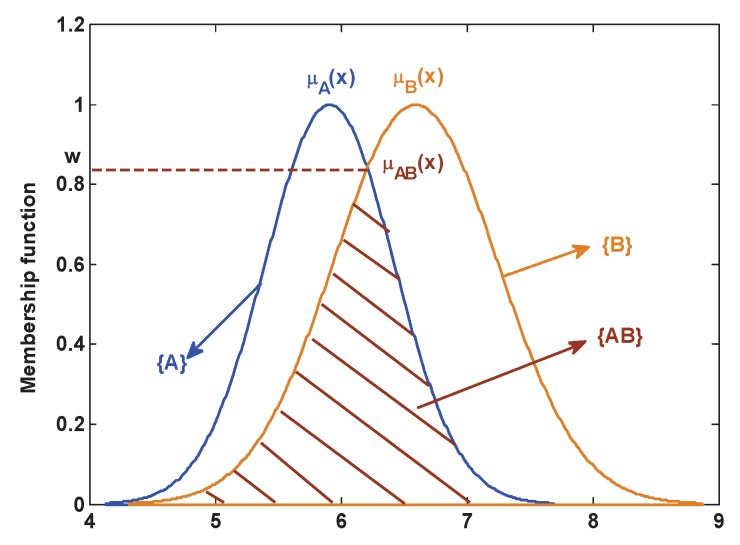
The modeling of the singleton subsect and compound subsect.

In this paper, the proposed method can automatically generate BBA of the singleton subsect and the compound subsect. This section mainly introduces how to construct the singleton subsect proposition and the compound subsect proposition. As shown in Figure 2, an attribute model of Gaussian type is denoted as a singleton subsect proposition, where $\mu_{A}\left(x\right)$ and $\mu_{B}\left(x\right)$ are all singleton subsect propositions. The compound subsect proposition is constructed by the overlap area of some membership functions of Gaussian type. The compound subsect proposition {AB} is noted as follows:

(12) $$\mu_{AB}\left(x\right)=min\left(\mu_{A}\left(x\right),\mu_{B}\left(x\right)\right)$$

where $w=sup\;min(\mu_{A}\left(x\right),\mu_{B}\left(x\right))$.

### 3.2. The Measurement of Similarity

In this paper, a nested structure similarity function sim is defined to represent the matching degree between the test sample and the attribute model. This structure can avoid the high conflict, to some extent. The similarity is measured using the following regulations:

(1) If there is only one intersection between the test sample and the attribute models, then this intersection is assigned to the corresponding singleton subset as the similarity between this test sample and the corresponding singleton subset model;

(2) If there is more than one intersection between the test sample and the attribute models, then the top value of the intersections is assigned to the corresponding singleton subset as the similarity; the low value of the intersections is assigned to the corresponding compound subset as the similarity.

**Figure 3:**
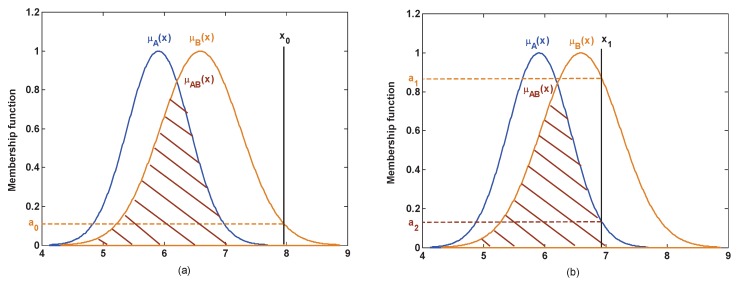
The measurement of similarity where the test model is a discrete value. (**a**) Only one intersection; (**b**) multiple intersections.

**Figure 4:**
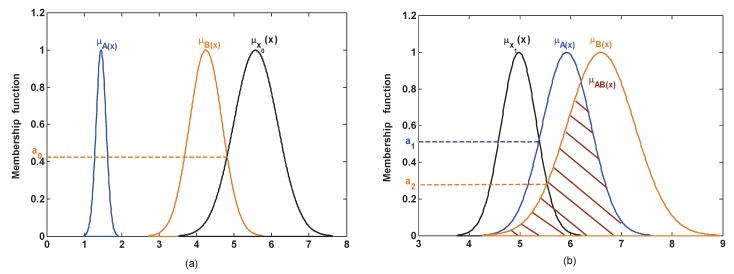
The measurement of similarity where the test model is a continuous value. (**a**) Only one intersection; (**b**) multiple intersections.

In the actual applications, the test model is covered by a different number of test data. For example, in the dataset, the test model is usually covered by one test data which, on account of its attribute value, is a fixed value, such as Iris. So this test model is the discrete value, as is the case for the test samples $x_{0}$ and $x_{1}$ which are shown in Figure 3. However, in a complex system, the attribute value is not a fixed value, for example, sensor data is not constant in sensor networks and is thus easily affected by the external environment. In such a case, the attribute is measured repeatedly by the sensor and the sensor data will be a set of values. Thus, this test model is covered by multiple test data, and is fuzzed into the membership function of Gaussian type, as is the case for the test models $\mu_{x_{0}}$ and $\mu_{x_{1}}$ which are shown in Figure 4. As shown in the analysis above, under the different application backgrounds, the measurement of the similarity is slightly different.

If the test model is covered by one test data, as shown in Figure 3a, for the test sample $x_{0}$, the similarity is measured by the above regulations (1):

$$sim\left(B\right)=\mu_{B}\left(x_{0}\right)=a_{0},$$

where $\mu_{B}$ is the membership functions of the singleton subsect propositions {B}. $x_{0}$ has no intersection with $\mu_{A}$ and $\mu_{AB}$, so sim(A)=sim(AB)=0.

As shown in Figure 3b, for the test sample $x_{1}$, the similarity is measured by the above regulations (2):

$$\begin{array}{c} sim\left(B\right)=\mu_{B}\left(x_{1}\right)=a_{1},\\ sim\left(AB\right)=\mu_{AB}\left(x_{1}\right)=a_{2}, \end{array}$$

where $\mu_{B}$ is the membership functions of the singleton subsect propositions {B}, and $\mu_{AB}$ is the membership functions of the compound subsect propositions {AB}.

Although $x_{1}$ has an intersection with $\mu_{A}$, this intersection is assigned to the compound subsect {AB} since it simultaneously locates in the overlapping portions between $\mu_{A}$ and $\mu_{B}$. Based on the above regulations (2), sim(A)=0 and $sim\left(AB\right)=a_{2}$.

If the test model is covered by multiple test data, then the model is first constructed using the membership function of Gaussian type, which is similar to the model of training sets. Then, as shown in Figure 4a, for the test model $\mu_{x_{0}}$, the similarity is measured by the above regulations (1):

$$sim\left(B\right)=a_{0},$$

where $\mu_{x_{0}}$ has no intersection with $\mu_{A}$ and $\mu_{AB}$, so sim(B)=sim(AB)=0.

As shown in Figure 4b, for the test model $\mu_{x_{1}}$, the similarity is measured by the above regulations (2):

$$sim\left(A\right)=a_{1},\;\;\;sim\left(AB\right)=a_{2},$$

where although $\mu_{x_{1}}$ has an intersection with $\mu_{\tilde{B}}$, this intersection is assigned to the compound subsect {AB} since it simultaneously locates in the overlapping portions between $\mu_{A}$ and $\mu_{B}$. Based on the regulations (2), sim(B)=0 and $sim\left(AB\right)=a_{2}$.

### 3.3. The Construction of Attribute Weights

In the multiclass classification, a comprehensive evaluation of each attribute needs to be made to obtain an objective result [47,48,49]. Given an attribute, if the similarity of some classes is high, that is, the overlap degree of the attribute models of these classes is large, then the ability to discriminate the difference of these classes of this attribute is weak. In this case, a false classification may easily occur based on this attribute, so the reliability of this attribute is low. Further, BBA generated from this attribute has a smaller contribution in multiclass classification. On the contrary, if the similarity of some classes is low, then the ability to discriminate the difference of these classes of this attribute is fine, so the reliability of this attribute is high. Further, BBA generated from this attribute has a larger contribution in multiclass classification. Thus, the effect of the attributes which provide a larger contribution should be enhanced and the effect of the attributes which provide a smaller contribution should be weakened to obtain more objective results. As analyzed above, the attribute weights are proposed and taken into account in this paper.

Suppose $\mu_{ij}\left(i=1,2,\cdots ,n;j=1,2,\cdots ,k\right)$ is the membership function of the *j*th attribute of the *i*th class; $\mu_{rj}^{*}\left(r=1,2,\cdots ,\frac{n^{2}-n}{2};j=1,2,\cdots ,k\right)$ is the generalized triangular fuzzy number model of the *r*th compound subsect proposition {AB} in the *j*th attribute; S(x) is the area of the *x*. Then the attribute weight $w_{j}\left(j=1,2,\cdots ,k\right)$ is proposed as follows:

(13) $$w_{j}=1-\frac{\sum_{r=1}^{\frac{n^{2}-n}{2}}S\left(\mu_{rj}^{*}\right)}{\sum_{i=1}^{n}S\left(\mu_{ij}\right)-\sum_{r=1}^{\frac{n^{2}-n}{2}}S\left(\mu_{rj}^{*}\right)}$$

where $w_{j}$ reveals that the larger the overlap degree, the larger the similarity, and the smaller the attribute weight.

### 3.4. The Construction of BBA

In D-S evidence theory, the closed world means that the FoD is complete; the open world means that the FoD is incomplete, namely, unknown propositions potentially exist outside of the given FoD. In the closed world, the FoD is complete, so the redundant BBA should be assigned to the universal set Θ, which is gained after using the attribute weights to correct the similarity sim. However, in the open world, an unknown proposition exists. In GET, BBA of the unknown proposition is determined based on the known propositions. Then, the attribute weights are also constructed by the model of the known propositions and used to correct the similarity of the known propositions. Finally, the redundant BBA is assigned to the universal set Θ. According to the analysis above, the determination of BBA in the closed world and the open world is proposed respectively.

#### 3.4.1. The Determination of BBA in the Closed World

In the closed world, BBA is determined by three steps. The similarity sim is generated in the first step, which is described in Section 3.2. In the second step, if ∑sim>1, the similarity is normalized; if ∑sim<1, the similarity is not normalized. The FoD is complete in the closed world, so where sim(∅)=0 is necessary. In the third step, BBA of each proposition is obtained as follows:

(14) $$\begin{array}{c} m(\emptyset )=0\\ m\left(A\right)=w_{j}\times sim_{j}\left(A\right),\;\;\;\left(A\neq \Theta \;and\;A\neq \emptyset \right)\\ m\left(\Theta \right)=1-\sum_{j=1}^{k}w_{j}sim_{j}\left(A\right) \end{array}$$

where $w_{j}$ is denoted as the *j*th attribute’s weight, and $sim_{j}$ is denoted as the similarity function of the *j*th attribute.

For example, in the frame of discernment Θ={a,b,c}, the similarity function $sim_{2}$ of attribute 2 is $sim_{2}\left(a\right)=0.6,sim_{2}\left(ab\right)=0.2$ and the attribute weigh $w_{2}=0.7$, then BBA is determined as follows:

$$\begin{array}{c} m_{2}\left(a\right)=w_{2}\times sim_{2}\left(a\right)=0.7\times 0.6=0.42,\\ m_{2}\left(ab\right)=w_{2}\times sim_{2}\left(ab\right)=0.7\times 0.2=0.14,\\ m_{2}\left(\Theta \right)=m_{2}\left(abc\right)=1-m_{2}\left(a\right)-m_{2}\left(ab\right)=1-0.42-0.14=0.44,\\ m_{2}\left(b\right)=m_{2}\left(c\right)=m_{2}\left(ac\right)=m_{2}\left(bc\right)=0. \end{array}$$

After determining BBA, BBAs of *k* attributes are combined k−1 times with Dempster’s combination rule and the final recognized result can be gained.

#### 3.4.2. The Determination of BBA in the Open World

In the open world, BBA is also determined by three steps. The similarity sim is generated in the first step, which is described in Section 3.2. The FoD is incomplete in the open world, so where sim(∅)=0 is not necessary. Hence, in the second step, if ∑sim>1, then the similarity is normalized and the similarity of the empty set sim(∅)=0; if ∑sim<1, then the similarity is not normalized and the similarity of the empty set sim(∅)=1−∑sim. In the third step, BBA of each proposition is obtained as follows:

(15) $$\begin{array}{c} m(\emptyset )=sim(\emptyset )\\ m\left(A\right)=w_{j}\times sim_{j}\left(A\right),\;\;\;\left(A\neq \Theta \;and\;A\neq \emptyset \right)\\ m\left(\Theta \right)=1-\sum_{j=1}^{k}w_{j}sim_{j}\left(A\right)-m\left(\emptyset \right) \end{array}$$

where $w_{j}$ is denoted as the *j*th attribute’s weight, and $sim_{j}$ is denoted as the similarity function of the *j*th attribute.

For example, in the frame of discernment Θ={a,b}, the similarity function $sim_{1}$ of attribute 1 is $sim_{1}\left(a\right)=0.6,sim_{1}\left(ab\right)=0.2$ and the attribute weigh $w_{1}=0.8$, then BBA is determined as follows. Firstly, the similarity of the empty set is obtained as:

$$sim_{1}\left(\emptyset \right)=1-\left(sim_{1}\left(a\right)+sim_{1}\left(ab\right)\right)=1-\left(0.6+0.2\right)=0.2.$$

Then BBA of each proposition is obtained as:

$$\begin{array}{c} m_{1}\left(\emptyset \right)=sim_{1}\left(\emptyset \right)=0.2,\\ m_{1}\left(a\right)=w_{1}\times sim_{1}\left(a\right)=0.8\times 0.6=0.48,\\ m_{1}\left(b\right)=w_{1}\times sim_{1}\left(b\right)=0.8\times 0=0,\\ m_{1}\left(\Theta \right)=m_{1}\left(ab\right)=1-m_{1}\left(\emptyset \right)-m_{1}\left(a\right)-m_{1}\left(b\right)=1-0.2-0.48-0=0.32. \end{array}$$

After determining BBA, BBAs of *k* attributes are combined k−1 times with the mGCR and the final recognized result can be gained.

## 4. Application Example

In this section, several experiments are performed both in the closed world and the open world respectively, to evaluate the validity and reasonability of the presented method. To better evaluate this method, an engineering example of fault diagnosis of a motor rotor is carried out. Note that the numbers from the following calculations are all unified as the four significant digits.

In addition, since the real-world data usually has stochastic nature, it is less advisable that the data with the highest recognition rate is selected for the experiment. Hence, in the following experiment, the training set and the test set are randomly selected, which makes the results more objective and credible.

### 4.1. An Example of Iris

The Iris dataset [50,51] contains three classes: Iris Setosa(S), Iris Versicolour(E) and Iris Virginica(V). Each class has 50 samples, and each sample contains four attributes: sepal length(SL), sepal width(SW), petal length(PL) and petal width(PW), respectively. Each of the four attributes is treated as an information source, and correspondingly there are three training sets and three test sets which are the same for the sample number. The data is obtained from the UCI repository of machine learning databases. (UCI Machine Learning Repository: http://archive.ics.uci.edu/ml/datasets/Iris.)

#### 4.1.1. Experiment in the Closed World

Thirty samples are randomly selected as training samples from three classes respectively, and the remaining 20 samples of each of the three classes are taken as the test samples. Then, the experiment is carried out as follows.

**Figure 5:**
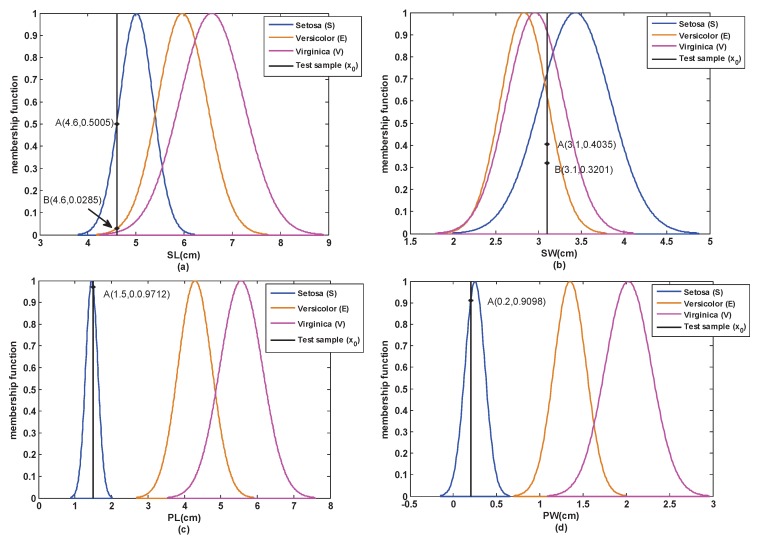
The models and similarity of each attribute in the closed world. (**a**) SL; (**b**) SW; (**c**) PL; (**d**) PW.

Step 1: According to Section 3.1, the attribute models of each attribute of the training samples are obtained and shown in Figure 5.

Step 2: A test sample (4.6,3.1,1.5,0.2) is selected from the test set of S. Then, based on Section 3.2, the similarity between this test sample and each attribute model is obtained, which is shown in Figure 5 and Table 1.

From Table 1, we can see that the similarity of the SW attribute does not coincide with the reality. This is because the SW attributes of the three classes are mutually overlapping, which is not easy to distinguish in this case.

Step 3: According to Equation (13), the attribute weights of the four attributes are obtained as follows:

$$\begin{array}{c} w_{SL}=1-\frac{1.0247}{3.6677-1.0247}=0.6123,\\ w_{SW}=1-\frac{0.7934}{2.2250-0.7934}=0.4458,\\ w_{PL}=1-\frac{0.2980}{3.0420-0.2980}=0.8914,\\ w_{PW}=1-\frac{0.0787}{1.4263-0.0787}=0.9416. \end{array}$$

Step 4: Based on Section 3.4, BBAs are determined as shown in Table 2.

Finally, four BBAs are combined three times with Dempster’s combination rule (Equation(4)), and the results are shown as follows:

m(S)=0.9714,m(E)=0,m(V)=0,m(SE)=0.0021,m(SV)=0.0009,m(EV)=0,m(SEV)=0.0256.

From the final BBA, it is clear that the test sample is classified into S class which coincides with the reality.

#### 4.1.2. Experiment in the Open World

To verify the presented method’s performance in the open world, the training samples are randomly selected only from two Iris classes which are randomly selected from the three classes, and the remaining class is taken as the unknown class. The test samples are randomly selected from each of the three classes. In the Iris dataset, each class has 50 samples. In this paper, 30 samples of two classes are randomly selected as training samples from S and E respectively; the remaining 20 samples of each of these two classes and the 20 samples which are randomly selected from V, are taken as the test samples. Then, the experiment is carried out as follows.

**Figure 6:**
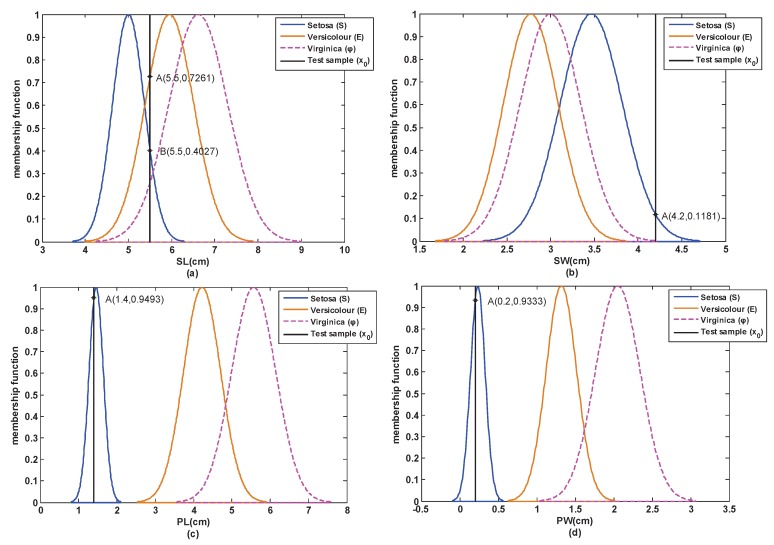
The models and similarity of each attribute in the open world.(**a**) SL; (**b**) SW; (**c**) PL; (**d**) PW.

Step 1: According to Section 3.1, the attribute model of each attribute of the training samples is obtained and shown in Figure 6.

Step 2: A test sample (5.5,4.2,1.4,0.2) is selected from the test set of S. Then, based on Section 3.2, the similarity between this test sample and each attribute model is obtained, which is shown in Figure 6 and Table 3.

From Table 3, we can see that the similarity of the PL attribute and the PW attribute coincides with the reality, but the similarity of the SL attribute and the SW attribute does not coincide with the reality. This is because the classes are not easy to distinguish if the overlap degree among the classes in the SL attribute and the SW attribute is large. Hence, the attribute weights are necessary when determining BBA.

Step 3: According to Equation (13), the attribute weights of four attributes are obtained as follows:

$$\begin{array}{c} w_{SL}=1-\frac{0.3605}{2.3381-0.3605}=0.8177,\\ w_{SW}=1-\frac{0.2504}{1.6729-0.2504}=0.8240,\\ w_{PL}=1-\frac{0}{1.6847-0}=1,\\ w_{PW}=1-\frac{0}{0.7362-0}=1. \end{array}$$

Step 4: Based on Section 3.4, BBAs are determined as shown in Table 4.

Finally, four BBAs are combined three times with the mGCR (Equation(8)), and the results are shown as follows:

m(S)=0.9980,m(E)=0.0012,m(SE)=0.0007,m(∅)=0.0001.

From the final BBA, it is clear that the test sample is classified into S class which coincides with the reality.

Similarly, a test sample (6.8,3.0,5.5,2.1) is selected from the test set of unknown class (V), then the final BBA is obtained as follows:

m(S)=0,m(E)=0,m(SE)=0,m(∅)=1,

where m(∅)=1 indicates that the possibility of this test sample outside the FoD is very large, which coincides with the reality.

### 4.2. Experiments on Three Datasets: Five-Fold Cross-Validation

To further evaluate the proposed method, this method is compared with three well-known classifiers: support vector machine with radial basis function (SVM-RBF), decision tree learner (REPTree) and Naive Bayesian (NB) both in the closed world and the open world.

Three datasets are experimented in this section, which are obtained from the UCI repository of machine learning databases [52]. (UCI Machine Learning Repository: http://archive.ics.uci.edu/ml/datasets.) Within this, the Iris dataset is perhaps the best known database found in the pattern recognition literature. The dataset contains three classes of 50 instances each, where each class refers to a type of iris plant. Each class contains four attributes. The Seeds dataset contains three different varieties of wheat: Kama (K), Rosa (R) and Canadian (C). Each variety has 70 samples, and each sample contains seven attributes. The Wine dataset was the result of a chemical analysis of wines grown in the same region in Italy but derived from three different cultivars. The analysis determined the quantities of 13 constituents found in each of the three types of wine. A summary of these datasets is shown in Table 5.

The comparison results of five-fold cross-validation are shown in Table 6, Table 7 and Table 8 respectively.

From the above experimental results, it is found that:

(1) The results in the closed world

Both the proposed method and the classical machine learning algorithms are efficient in the closed world. From the first four lines of Table 6, Table 7 and Table 8, we can find that the proposed method obtains competitive performances with respect to machine learning algorithms. For example, in the first four lines of Table 7, the average recognition rate of Seeds of our method is 90.57%, NB is 78.09%, REPTree is 89.49% and SVM-RBF is 90.21%.

(2) The results in the open world

From the latter 12 lines of Table 6, Table 7 and Table 8, we can find that the average recognition rate of the empty set ∅ is 0 by the classical machine learning classifiers. It means that the “unknown” class is definitely misclassified by the classical machine learning classifiers. These standard machine learning classification algorithms cannot work in an open world environment.

In contrast, the “unknown” class in an open world environment can be classified by the proposed method. For example, in Table 8, the average recognition rate of the empty set ∅ of wine is 89.33%, 93.78% and 91.62% respectively in the frame of discernment of {A,B}, {A,C} and {B,C}. Therefore, our method is still effective in an open world.

By summarizing (1) the results in the closed world and (2) the results in the open world, it can be found that both the proposed method and the classical machine learning algorithms are efficient in the closed world. However, the classical machine learning algorithms cannot work in an open world. In contrast, our method is still effective in an open world, which is the advantage of our method compared with the classical machine learning algorithms.

### 4.3. An Example of Fault Diagnosis

Suppose there are three types of fault in a motor rotor, which are noted as $F=\left\{F_{1},F_{2},F_{3}\right\}=\left\{rotorunbalance,rotormisalignment,Pedestallooseness\right\}$. Three vibration acceleration sensors and a vibration displacement sensor are placed in different installation positions to collect the vibration signal. Vibration displacement and acceleration vibration frequency amplitudes at the frequencies of 1X, 2X and 3X are taken as the fault feature variables. At the same time interval, each fault feature of each fault is continuously observed 40 times which is taken as a group of observations. In this paper, a total of five groups are measured in each fault feature of each fault. For example, five groups of observations of the fault feature variables of 1X of the rotor unbalance are shown in Table 9.

The example is carried out by the proposed method and three well-known classifiers: support vector machine with radial basis function (SVM-RBF), decision tree learner (REPTree) and Naive Bayesian (NB), respectively. The comparison results of the five-fold cross-validation are shown in Table 10.

The above experimental results demonstrate the validity of the proposed method. The advantages of the proposed method are discussed and concluded as follows:

- The weights of the attributes have been considered in the proposed BBA generation method. Because of that, the generated BBA could reflect the difference of the importance between different attributes which have different classification ability. If the classification ability of each attribute is similar, then the result obtained by using the proposed method is basically identical with such a method that does not consider attributes’ weights. However, if attributes have different classification ability, then the proposed method has better performance since it has considered the weight of each attribute.
- The weight of each attribute is totally determined based on the discrimination of the attribute, but without relying on other information. In many existing approaches, the weights of attributes are either from an externally given credibility of sensors or mutual support degree of different attributes. In this paper, the weight of every attribute is determined just by the attribute’s discrimination, before the amendment of the generated BBA is implemented. This approach is completely a data-driven solution, which makes the results more objective and credible.
- The proposed BBA generation method is efficient simultaneously in the closed world and open world. At present, many existing BBA generation approaches only consider the situation of the closed world. In contrast, by introducing generalized evidence theory, the incomplete information about the frame of discernment is taken into consideration in the initial phase of the modelling of uncertain information. Therefore, it is more reasonable and realistic.
- The proposed method is simple and can be easily used in many practical applications. In addition, it is based on the normal distribution assumption and can be easily changed to other forms to reflect the feature of training data much more realistically. Therefore, the proposed method is flexible and easily extensible.

However, compared to the traditional BBA method, namely the method that does not consider the attribute weights, the approach of weighted BBA has complicated calculations. However, considering that the performance is better after using the attribute weights, the increased computational complexity is acceptable to some extent.

## 5. Conclusions

In the application of soft sensors data fusion, based on evidence theory, how to determine BBA is an open issue. In this paper, a new method is presented to determine BBA in a closed or open world environment. Within the proposed BBA determination method, all data are separated into a training set and test set, and the attributes of each sample are seen as soft sensors. At first, according to the training samples, each attribute’s Gaussian model is built. Then, for every test sample, by considering the similarity and the attribute weights, a BBA is derived to express the test sample. Finally, according to the derived BBA, the class of the corresponding test sample is determined.

The advantages of the proposed BBA determination method include the following aspects. At first, the weights of attributes, which reflect the reliability of information sources, have been considered in the process of BBA generation. Moreover, it is compatible with the method which does not consider the attribute weights. Second, since the generalized evidence theory has been used in this work, the proposed method is efficient not only in the closed world but also in the open world. Many previous BBA determination methods have only been able to deal with the situation of the closed world. Third, the whole process is completely data-driven, which makes the results more objective. The proposed method can be easily used in many practical applications, for example, classification and fault diagnosis. A number of simulation experiments show that the proposed method is effective. However, some shortcomings still exist in the proposed method. Mainly, the amount of computation of the proposed method is increased since the weights of attributes have been considered. Besides, the proposed method relies on a proper training set, which requires a certain size of data samples.

In the future, we will research further the following problems. Firstly, we will try to apply the proposed method to more practical applications, and import a pre-judging procedure to determine whether the attribute weights need to be considered to reduce the computational complexity. Furthermore, the possible dependencies among the attributes of the actual targets will be considered to improved the proposed method.

## Figures and Tables

**Table 1:** Similarity of Iris in the closed world.

Attributes	Similarity						
	sim(S)	sim(E)	sim(V)	sim(SE)	sim(SV)	sim(EV)	sim(SEV)
**SL**	0.5005	0	0	0.0285	0	0	0
**SW**	0	0	0.4035	0	0.3201	0	0
**PL**	0.9712	0	0	0	0	0	0
**PW**	0.9098	0	0	0	0	0	0

**Table 2:** BBA of Iris in the closed world.

Attributes	BBA						
	m(S)	m(E)	m(V)	m(SE)	m(SV)	m(EV)	m(SEV)
**SL**	0.3046	0	0	0.0515	0	0	0.6439
**SW**	0.1665	0	0	0	0.0283	0	0.8052
**PL**	0.7442	0	0	0	0	0	0.2558
**PW**	0.8067	0	0	0	0	0	0.1933

**Table 3:** Similarity of Iris in the open world.

Attributes	Similarity		
	sim(S)	sim(E)	sim(SE)
**SL**	0	0.7261	0.4027
**SW**	0.1181	0	0
**PL**	0.9493	0	0
**PW**	0.9333	0	0

**Table 4:** BBA of Iris in the open world.

Attributes	BBA			
	m(S)	m(E)	m(SE)	m(∅)
**SL**	0	0.5937	0.3678	0.0385
**SW**	0.0973	0	0.4513	0.4513
**PL**	0.9493	0	0.0253	0.0253
**PW**	0.9333	0	0.0333	0.0333

**Table 5:** General information about the real datasets.

Dataset	#Instance	#Class	#Attribute
**Iris**	150	3	4
**Seed**	210	3	7
**Wine**	178	3	13

**Table 6:** The comparison results of Iris.

Cases	Frame	Methods	Classes			Overall Average
			Setosa (S)	Versicolour (E)	Virginica (V)	
**Closed world**	{S,E,V}	SVM-RBF	100.00%	94.30%	96.31%	96.87%
		REPTree	100.00%	92.38%	92.65%	95.01%
		NB	100%	91.62%	94.53%	95.38%
		Our method	99.00%	93.00%	95.00%	95.67%
**Open world**	{S,E}	SVM-RBF	100.00%	100.00%	0 (V=∅)	66.67%
		REPTree	100.00%	100.00%	0 (V=∅)	66.67%
		NB	100.00%	100.00%	0 (V=∅)	66.67%
		Our method	92.00%	88.00%	84.00% (V=∅)	88%
	{S,V}	SVM-RBF	100.00%	0 (E=∅)	100.00%	66.67%
		REPTree	99.41%	0 (E=∅)	99.67%	66.36%
		NB	100%	0 (E=∅)	100.00%	66.67%
		Our method	84.00%	80.00% (E=∅)	90.00%	84.67%
	{E,V}	SVM-RBF	0 (S=∅)	95.53%	97.89%	64.48%
		REPTree	0 (S=∅)	93.11%	90.61%	61.24%
		NB	0 (S=∅)	92.51%	94.89%	62.47%
		Our method	100.00% (S=∅)	82.00%	84.00%	88.67%

**Table 7:** The comparison results of Seeds.

Cases	Frame	Methods	Classes			Overall Average
			Kama (K)	Rosa (R)	Canadian (C)	
**Closed world**	{K,R,C}	SVM-RBF	82.14%	94.41%	94.08%	90.21%
		REPTree	84.32%	92.33%	91.82%	89.49%
		NB	76.03%	69.81%	88.42%	78.09%
		Our method	87.71%	93.43%	90.57%	90.57%
**Open world**	{K,R}	SVM-RBF	93.07%	93.58%	0 (C=∅)	62.21%
		REPTree	93.57%	95.43%	0 (C=∅)	63.00%
		NB	78.04%	77.22%	0 (C=∅)	51.75%
		Our method	85.71%	82.86%	88.57% (C=∅)	85.71%
	{K,C}	SVM-RBF	89.84%	0 (R=∅)	93.61%	61.15%
		REPTree	89.44%	0 (R=∅)	91.04%	60.16%
		NB	83.67%	0 (R=∅)	87.66%	57.11%
		Our method	80.00%	88.57% (R=∅)	95.71%	88.09%
	{R,C}	SVM-RBF	0 (K=∅)	100%	100%	66.67%
		REPTree	0 (K=∅)	98.81%	99.53%	66.11%
		NB	0 (K=∅)	91.91%	89.12%	60.34%
		Our method	84.29% (K=∅)	91.43%	84.29%	86.67%

**Table 8:** The comparison results of Wine.

Cases	Frame	Methods	Classes			Overall Average
			A	B	C	
**Closed world**	{A,B,C}	SVM-RBF	6.86%	99.93%	4.75%	37.18%
		REPTree	91.43%	88.06%	91.58%	90.36%
		NB	88.36%	82.88%	84.58%	85.27%
		Our method	89.85%	95.90%	95.78%	93.84%
**Open world**	{A,B}	SVM-RBF	7.36%	99.36%	0 (C=∅)	35.68%
		REPTree	95.35%	95.87%	0 (C=∅)	63.74%
		NB	88.07%	93.19%	0 (C=∅)	60.42%
		Our method	84.85%	88.95%	89.33% (C=∅)	87.71%
	{A,C}	SVM-RBF	100.00%	0 (B=∅)	6.91%	35.64%
		REPTree	99.29%	0 (B=∅)	99.17%	66.15%
		NB	86.93%	0 (B=∅)	95.92%	60.95%
		Our method	95.00%	93.78% (B=∅)	81.90%	90.23%
	{B,C}	SVM-RBF	0 (A=∅)	100%	5.25%	35.08%
		REPTree	0 (A=∅)	94.31%	91.92%	62.08%
		NB	0 (A=∅)	84.88%	88.00%	57.63%
		Our method	91.62% (A=∅)	96.00%	79.85%	89.16%

**Table 9:** Five groups of observations of 1X of the rotor unbalance [53].

Groups	Observations									
**X11**	0.1663	0.1590	0.1568	0.1485	0.1723	0.2006	0.1903	0.1908	0.1986	0.1843
	0.1785	0.1610	0.1579	0.1511	0.1532	0.1647	0.1628	0.1646	0.1634	0.1642
	0.1648	0.1640	0.1674	0.0661	0.1659	0.1650	0.1633	0.1632	0.1604	0.1542
	0.1555	0.1562	0.1540	0.1564	0.1557	0.1542	0.1546	0.1571	0.1537	0.1536
**X12**	0.154	0.1518	0.1537	0.1548	0.1542	0.1538	0.1545	0.1537	0.1571	0.1560
	0.1584	0.1552	0.1586	0.1574	0.1569	0.1565	0.1551	0.1585	0.1585	0.1593
	0.1548	0.1558	0.1547	0.1593	0.1532	0.1632	0.1575	0.159	0.1594	0.1541
	0.165	0.1674	0.1651	0.1604	0.1787	0.1818	0.1820	0.1656	0.1658	0.1644
**X13**	0.1647	0.1647	0.1654	0.1651	0.1656	0.1653	0.1652	0.1652	0.1648	0.1649
	0.1653	0.1650	0.1650	0.1652	0.1653	0.1652	0.1648	0.1647	0.1646	0.1645
	0.1651	0.1652	0.1652	0.1649	0.1650	0.1643	0.1640	0.1639	0.1641	0.1633
	0.1632	0.1629	0.1630	0.1630	0.1634	0.1631	0.1634	0.1629	0.1632	0.1629
**X14**	0.1630	0.1629	0.1627	0.1626	0.1622	0.1624	0.1627	0.1618	0.1614	0.1617
	0.1621	0.1615	0.1618	0.1611	0.1614	0.1610	0.1612	0.1611	0.1616	0.1612
	0.1612	0.1613	0.1623	0.1616	0.1621	0.1613	0.1611	0.1610	0.1610	0.1613
	0.1615	0.1616	0.1618	0.1616	0.1614	0.1612	0.1606	0.1614	0.1619	0.1614
**X15**	0.1609	0.1610	0.1612	0.1615	0.1609	0.1606	0.1604	0.1606	0.1605	0.1601
	0.1604	0.1608	0.1610	0.1603	0.1599	0.1601	0.1602	0.1599	0.1598	0.1598
	0.1598	0.1596	0.1595	0.1593	0.1594	0.1598	0.1596	0.1597	0.1595	0.1593
	0.1598	0.1596	0.1597	0.1595	0.1593	0.1577	0.1580	0.1576	0.1577	0.1579

**Table 10:** The comparison results of the fault diagnosis.

Cases	Frame	Methods	Classes			Overall Average
			$F_{1}$	$F_{2}$	$F_{3}$	
**Closed world**	{$F_{1},F_{2},F_{3}$}	SVM-RBF	94.15%	92.86%	100%	95.67%
		REPTree	99.05%	98.68%	99.78%	99.17%
		NB	98.05%	96.94%	100%	98.33%
		Our method	99.50%	98.50%	100%	99.33%
**Open world**	{$F_{1},F_{2}$}	SVM-RBF	99.14%	98.49%	0($F_{3}$=∅)	65.88%
		REPTree	99.66%	100%	0($F_{3}$=∅)	66.55%
		NB	98.86%	99.48%	0($F_{3}$=∅)	66.11%
		Our method	96.00%	90.00%	100%($F_{3}$=∅)	95.33%
	{$F_{1},F_{3}$}	SVM-RBF	99.86%	0($F_{2}$=∅)	100%	66.62%
		REPTree	99.76%	0($F_{2}$=∅)	100%	66.59%
		NB	99.99%	0($F_{2}$=∅)	100%	66.66%
		Our method	96.50%	100%($F_{2}$=∅)	94%	96.83%
	{$F_{2},F_{3}$}	SVM-RBF	0($F_{1}$=∅)	99.38%	100%	66.46%
		REPTree	0($F_{1}$=∅)	99.70%	99.78%	66.49%
		NB	0($F_{1}$=∅)	99.41%	100%	66.47%
		Our method	100%($F_{1}$=∅)	91.00%	93.00%	94.67%
